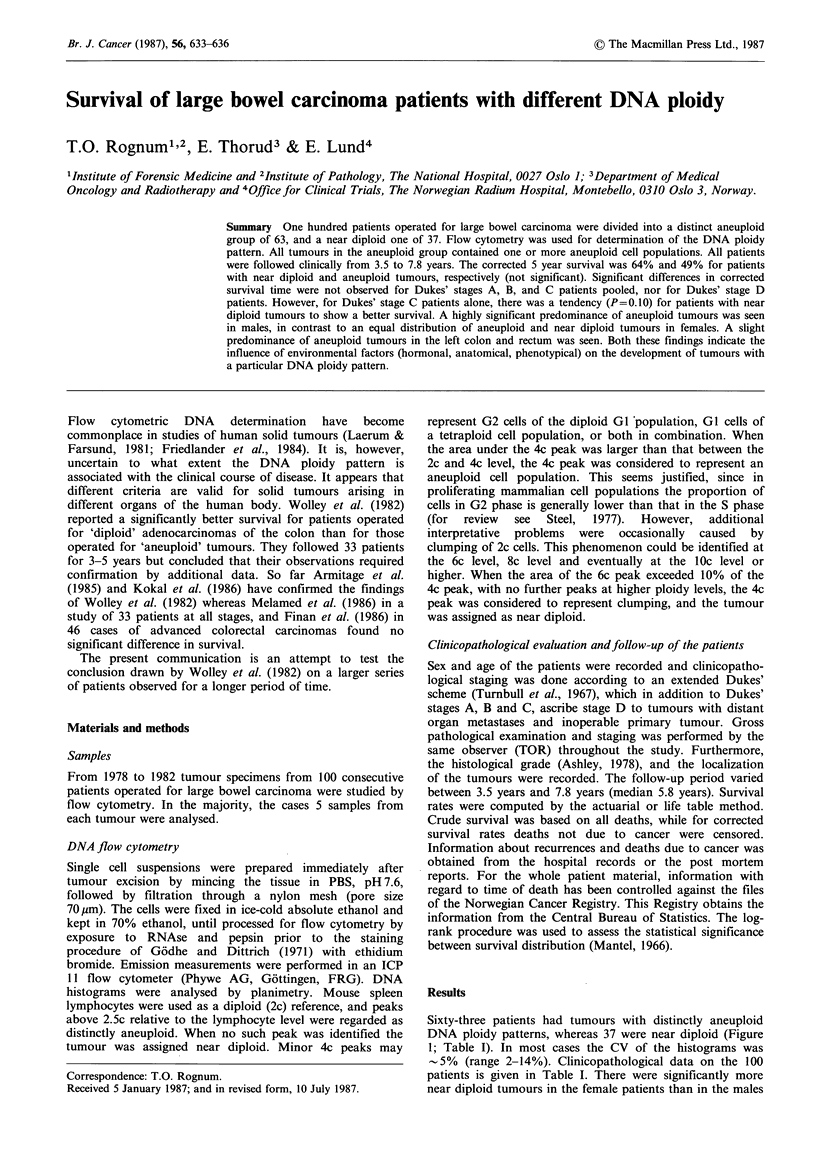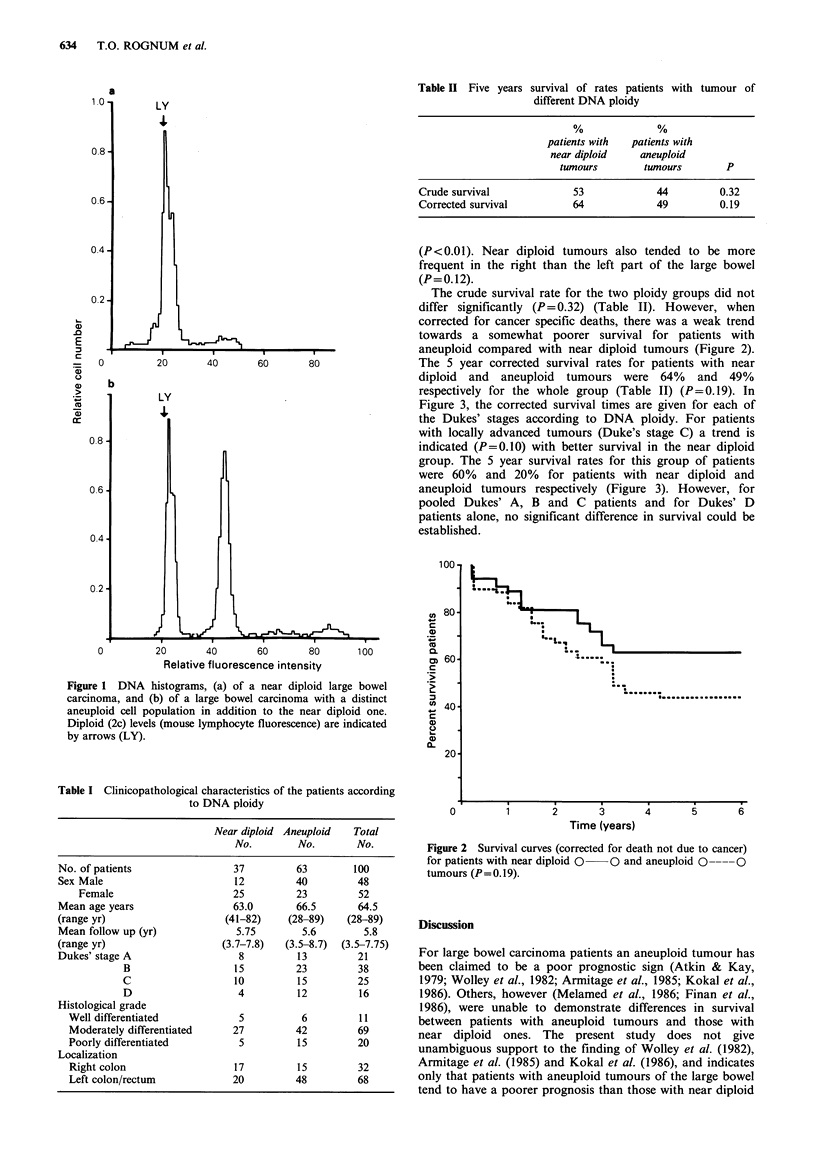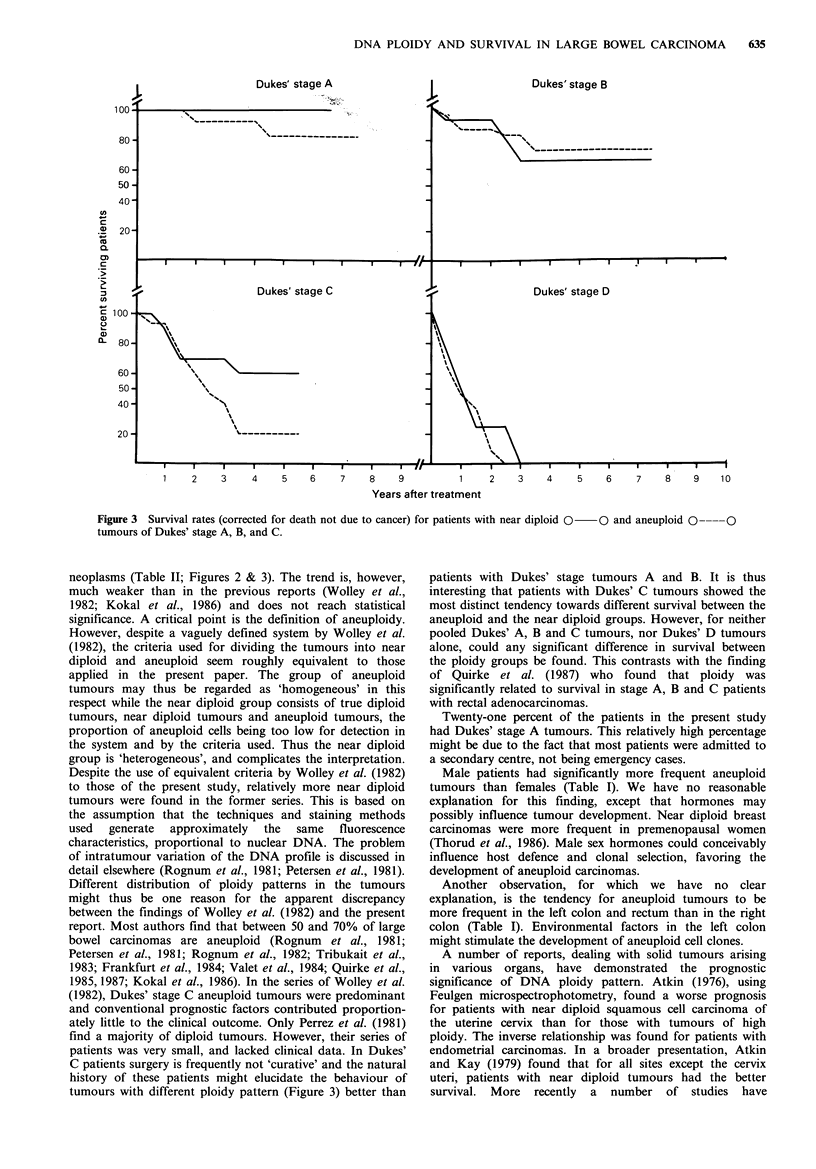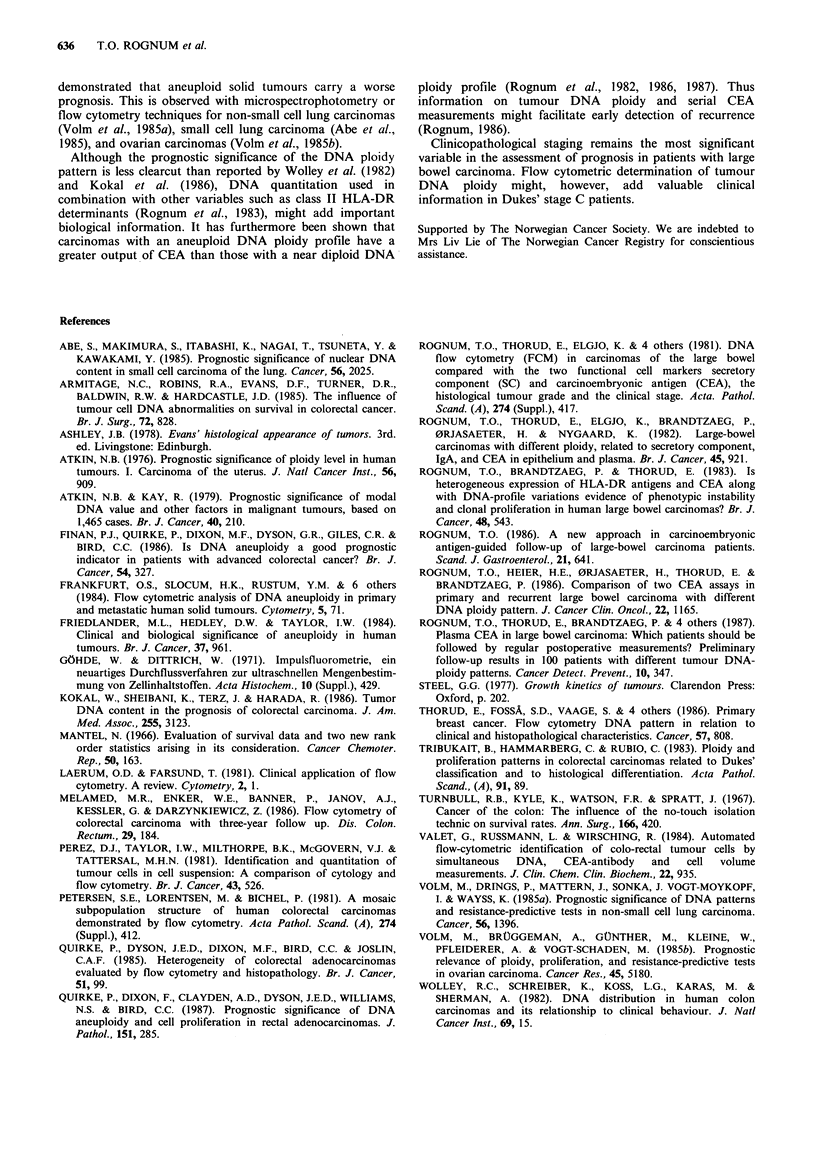# Survival of large bowel carcinoma patients with different DNA ploidy.

**DOI:** 10.1038/bjc.1987.257

**Published:** 1987-11

**Authors:** T. O. Rognum, E. Thorud, E. Lund

**Affiliations:** Institute of Forensic Medicine, National Hospital, Oslo, Norway.

## Abstract

One hundred patients operated for large bowel carcinoma were divided into a distinct aneuploid group of 63, and a near diploid one of 37. Flow cytometry was used for determination of the DNA ploidy pattern. All tumours in the aneuploid group contained one or more aneuploid cell populations. All patients were followed clinically from 3.5 to 7.8 years. The corrected 5 year survival was 64% and 49% for patients with near diploid and aneuploid tumours, respectively (not significant). Significant differences in corrected survival time were not observed for Dukes' stages A, B, and C patients pooled, nor for Dukes' stage D patients. However, for Dukes' stage C patients alone, there was a tendency (P = 0.10) for patients with near diploid tumours to show a better survival. A highly significant predominance of aneuploid tumours was seen in males, in contrast to an equal distribution of aneuploid and near diploid tumours in females. A slight predominance of aneuploid tumours in the left colon and rectum was seen. Both these findings indicate the influence of environmental factors (hormonal, anatomical, phenotypical) on the development of tumours with a particular DNA ploidy pattern.


Br. J. Cancer (1987), 56, 633–636 © The Macmillan Press Ltd., 1987

### Survival of large bowel carcinoma patients with different DNA ploidy

T.O. Rognum[1,2], E. Thorud[3] & E. Lund[4]

[1]*Institute of Forensic Medicine and* [2]*Institute of Pathology, The National Hospital, 0027 Oslo 1;* [3]*Department of Medical Oncology and Radiotherapy and* [4]*Office for Clinical Trials, The Norwegian Radium Hospital, Montebello, 0310 Oslo 3, Norway.*

**Summary** One hundred patients operated for large bowel carcinoma were divided into a distinct aneuploid group of 63, and a near diploid one of 37. Flow cytometry was used for determination of the DNA ploidy pattern. All tumours in the aneuploid group contained one or more aneuploid cell populations. All patients were followed clinically from 3.5 to 7.8 years. The corrected 5 year survival was 64% and 49% for patients with near diploid and aneuploid tumours, respectively (not significant). Significant differences in corrected survival time were not observed for Dukes' stages A, B, and C patients pooled, nor for Dukes' stage D patients. However, for Dukes' stage C patients alone, there was a tendency ($P=0.10$) for patients with near diploid tumours to show a better survival. A highly significant predominance of aneuploid tumours was seen in males, in contrast to an equal distribution of aneuploid and near diploid tumours in females. A slight predominance of aneuploid tumours in the left colon and rectum was seen. Both these findings indicate the influence of environmental factors (hormonal, anatomical, phenotypical) on the development of tumours with a particular DNA ploidy pattern.

Flow cytometric DNA determination have become commonplace in studies of human solid tumours (Laerum & Farsund, 1981; Friedlander *et al.*, 1984). It is, however, uncertain to what extent the DNA ploidy pattern is associated with the clinical course of disease. It appears that different criteria are valid for solid tumours arising in different organs of the human body. Wolley *et al.* (1982) reported a significantly better survival for patients operated for 'diploid' adenocarcinomas of the colon than for those operated for 'aneuploid' tumours. They followed 33 patients for 3–5 years but concluded that their observations required confirmation by additional data. So far Armitage *et al.* (1985) and Kokal *et al.* (1986) have confirmed the findings of Wolley *et al.* (1982) whereas Melamed *et al.* (1986) in a study of 33 patients at all stages, and Finan *et al.* (1986) in 46 cases of advanced colorectal carcinomas found no significant difference in survival.

The present communication is an attempt to test the conclusion drawn by Wolley *et al.* (1982) on a larger series of patients observed for a longer period of time.

### Materials and methods

### *Samples*

From 1978 to 1982 tumour specimens from 100 consecutive patients operated for large bowel carcinoma were studied by flow cytometry. In the majority, the cases 5 samples from each tumour were analysed.

### *DNA flow cytometry*

Single cell suspensions were prepared immediately after tumour excision by mincing the tissue in PBS, pH 7.6, followed by filtration through a nylon mesh (pore size 70 $\mu$m). The cells were fixed in ice-cold absolute ethanol and kept in 70% ethanol, until processed for flow cytometry by exposure to RNAse and pepsin prior to the staining procedure of Gödhe and Dittrich (1971) with ethidium bromide. Emission measurements were performed in an ICP 11 flow cytometer (Phywe AG, Göttingen, FRG). DNA histograms were analysed by planimetry. Mouse spleen lymphocytes were used as a diploid (2c) reference, and peaks above 2.5c relative to the lymphocyte level were regarded as distinctly aneuploid. When no such peak was identified the tumour was assigned near diploid. Minor 4c peaks may represent G2 cells of the diploid G1 population, G1 cells of a tetraploid cell population, or both in combination. When the area under the 4c peak was larger than that between the 2c and 4c level, the 4c peak was considered to represent an aneuploid cell population. This seems justified, since in proliferating mammalian cell populations the proportion of cells in G2 phase is generally lower than that in the S phase (for review see Steel, 1977). However, additional interpretative problems were occasionally caused by clumping of 2c cells. This phenomenon could be identified at the 6c level, 8c level and eventually at the 10c level or higher. When the area of the 6c peak exceeded 10% of the 4c peak, with no further peaks at higher ploidy levels, the 4c peak was considered to represent clumping, and the tumour was assigned as near diploid.

### *Clinicopathological evaluation and follow-up of the patients*

Sex and age of the patients were recorded and clinicopathological staging was done according to an extended Dukes' scheme (Turnbull *et al.*, 1967), which in addition to Dukes' stages A, B and C, ascribe stage D to tumours with distant organ metastases and inoperable primary tumour. Gross pathological examination and staging was performed by the same observer (TOR) throughout the study. Furthermore, the histological grade (Ashley, 1978), and the localization of the tumours were recorded. The follow-up period varied between 3.5 years and 7.8 years (median 5.8 years). Survival rates were computed by the actuarial or life table method. Crude survival was based on all deaths, while for corrected survival rates deaths not due to cancer were censored. Information about recurrences and deaths due to cancer was obtained from the hospital records or the post mortem reports. For the whole patient material, information with regard to time of death has been controlled against the files of the Norwegian Cancer Registry. This Registry obtains the information from the Central Bureau of Statistics. The log-rank procedure was used to assess the statistical significance between survival distribution (Mantel, 1966).

### Results

Sixty-three patients had tumours with distinctly aneuploid DNA ploidy patterns, whereas 37 were near diploid (Figure 1; Table I). In most cases the CV of the histograms was ~5% (range 2–14%). Clinicopathological data on the 100 patients is given in Table I. There were significantly more near diploid tumours in the female patients than in the males

Correspondence: T.O. Rognum.
Received 5 January 1987; and in revised form, 10 July 1987.

**Figure 1** DNA histograms, (a) of a near diploid large bowel carcinoma, and (b) of a large bowel carcinoma with a distinct aneuploid cell population in addition to the near diploid one. Diploid (2c) levels (mouse lymphocyte fluorescence) are indicated by arrows (LY).

**Table I** Clinicopathological characteristics of the patients according to DNA ploidy

| | *Near diploid No.* | *Aneuploid No.* | *Total No.* |
|---|---|---|---|
| No. of patients | 37 | 63 | 100 |
| Sex Male | 12 | 40 | 48 |
| Female | 25 | 23 | 52 |
| Mean age years | 63.0 | 66.5 | 64.5 |
| (range yr) | (41–82) | (28–89) | (28–89) |
| Mean follow up (yr) | 5.75 | 5.6 | 5.8 |
| (range yr) | (3.7–7.8) | (3.5–8.7) | (3.5–7.75) |
| Dukes' stage A | 8 | 13 | 21 |
| B | 15 | 23 | 38 |
| C | 10 | 15 | 25 |
| D | 4 | 12 | 16 |
| Histological grade | | | |
| Well differentiated | 5 | 6 | 11 |
| Moderately differentiated | 27 | 42 | 69 |
| Poorly differentiated | 5 | 15 | 20 |
| Localization | | | |
| Right colon | 17 | 15 | 32 |
| Left colon/rectum | 20 | 48 | 68 |

**Table II** Five years survival of rates patients with tumour of different DNA ploidy

| | *% patients with near diploid tumours* | *% patients with aneuploid tumours* | *P* |
|---|---|---|---|
| Crude survival | 53 | 44 | 0.32 |
| Corrected survival | 64 | 49 | 0.19 |

($P<0.01$). Near diploid tumours also tended to be more frequent in the right than the left part of the large bowel ($P=0.12$).

The crude survival rate for the two ploidy groups did not differ significantly ($P=0.32$) (Table II). However, when corrected for cancer specific deaths, there was a weak trend towards a somewhat poorer survival for patients with aneuploid compared with near diploid tumours (Figure 2). The 5 year corrected survival rates for patients with near diploid and aneuploid tumours were 64% and 49% respectively for the whole group (Table II) ($P=0.19$). In Figure 3, the corrected survival times are given for each of the Dukes' stages according to DNA ploidy. For patients with locally advanced tumours (Duke's stage C) a trend is indicated ($P=0.10$) with better survival in the near diploid group. The 5 year survival rates for this group of patients were 60% and 20% for patients with near diploid and aneuploid tumours respectively (Figure 3). However, for pooled Dukes' A, B and C patients and for Dukes' D patients alone, no significant difference in survival could be established.

**Figure 2** Survival curves (corrected for death not due to cancer) for patients with near diploid O——O and aneuploid O----O tumours ($P=0.19$).

### Discussion

For large bowel carcinoma patients an aneuploid tumour has been claimed to be a poor prognostic sign (Atkin & Kay, 1979; Wolley *et al.*, 1982; Armitage *et al.*, 1985; Kokal *et al.*, 1986). Others, however (Melamed *et al.*, 1986; Finan *et al.*, 1986), were unable to demonstrate differences in survival between patients with aneuploid tumours and those with near diploid ones. The present study does not give unambiguous support to the finding of Wolley *et al.* (1982), Armitage *et al.* (1985) and Kokal *et al.* (1986), and indicates only that patients with aneuploid tumours of the large bowel tend to have a poorer prognosis than those with near diploid

**Figure 3** Survival rates (corrected for death not due to cancer) for patients with near diploid O——O and aneuploid O----O tumours of Dukes' stage A, B, and C.

neoplasms (Table II; Figures 2 & 3). The trend is, however, much weaker than in the previous reports (Wolley *et al.*, 1982; Kokal *et al.*, 1986) and does not reach statistical significance. A critical point is the definition of aneuploidy. However, despite a vaguely defined system by Wolley *et al.* (1982), the criteria used for dividing the tumours into near diploid and aneuploid seem roughly equivalent to those applied in the present paper. The group of aneuploid tumours may thus be regarded as 'homogeneous' in this respect while the near diploid group consists of true diploid tumours, near diploid tumours and aneuploid tumours, the proportion of aneuploid cells being too low for detection in the system and by the criteria used. Thus the near diploid group is 'heterogeneous', and complicates the interpretation. Despite the use of equivalent criteria by Wolley *et al.* (1982) to those of the present study, relatively more near diploid tumours were found in the former series. This is based on the assumption that the techniques and staining methods used generate approximately the same fluorescence characteristics, proportional to nuclear DNA. The problem of intratumour variation of the DNA profile is discussed in detail elsewhere (Rognum *et al.*, 1981; Petersen *et al.*, 1981). Different distribution of ploidy patterns in the tumours might thus be one reason for the apparent discrepancy between the findings of Wolley *et al.* (1982) and the present report. Most authors find that between 50 and 70% of large bowel carcinomas are aneuploid (Rognum *et al.*, 1981; Petersen *et al.*, 1981; Rognum *et al.*, 1982; Tribukait *et al.*, 1983; Frankfurt *et al.*, 1984; Valet *et al.*, 1984; Quirke *et al.*, 1985, 1987; Kokal *et al.*, 1986). In the series of Wolley *et al.* (1982), Dukes' stage C aneuploid tumours were predominant and conventional prognostic factors contributed proportionately little to the clinical outcome. Only Perrez *et al.* (1981) find a majority of diploid tumours. However, their series of patients was very small, and lacked clinical data. In Dukes' C patients surgery is frequently not 'curative' and the natural history of these patients might elucidate the behaviour of tumours with different ploidy pattern (Figure 3) better than patients with Dukes' stage tumours A and B. It is thus interesting that patients with Dukes' C tumours showed the most distinct tendency towards different survival between the aneuploid and the near diploid groups. However, for neither pooled Dukes' A, B and C tumours, nor Dukes' D tumours alone, could any significant difference in survival between the ploidy groups be found. This contrasts with the finding of Quirke *et al.* (1987) who found that ploidy was significantly related to survival in stage A, B and C patients with rectal adenocarcinomas.

Twenty-one percent of the patients in the present study had Dukes' stage A tumours. This relatively high percentage might be due to the fact that most patients were admitted to a secondary centre, not being emergency cases.

Male patients had significantly more frequent aneuploid tumours than females (Table I). We have no reasonable explanation for this finding, except that hormones may possibly influence tumour development. Near diploid breast carcinomas were more frequent in premenopausal women (Thorud *et al.*, 1986). Male sex hormones could conceivably influence host defence and clonal selection, favoring the development of aneuploid carcinomas.

Another observation, for which we have no clear explanation, is the tendency for aneuploid tumours to be more frequent in the left colon and rectum than in the right colon (Table I). Environmental factors in the left colon might stimulate the development of aneuploid cell clones.

A number of reports, dealing with solid tumours arising in various organs, have demonstrated the prognostic significance of DNA ploidy pattern. Atkin (1976), using Feulgen microspectrophotometry, found a worse prognosis for patients with near diploid squamous cell carcinoma of the uterine cervix than for those with tumours of high ploidy. The inverse relationship was found for patients with endometrial carcinomas. In a broader presentation, Atkin and Kay (1979) found that for all sites except the cervix uteri, patients with near diploid tumours had the better survival. More recently a number of studies have demonstrated that aneuploid solid tumours carry a worse prognosis. This is observed with microspectrophotometry or flow cytometry techniques for non-small cell lung carcinomas (Volm *et al.*, 1985*a*), small cell lung carcinoma (Abe *et al.*, 1985), and ovarian carcinomas (Volm *et al.*, 1985*b*).

Although the prognostic significance of the DNA ploidy pattern is less clearcut than reported by Wolley *et al.* (1982) and Kokal *et al.* (1986), DNA quantitation used in combination with other variables such as class II HLA-DR determinants (Rognum *et al.*, 1983), might add important biological information. It has furthermore been shown that carcinomas with an aneuploid DNA ploidy profile have a greater output of CEA than those with a near diploid DNA ploidy profile (Rognum *et al.*, 1982, 1986, 1987). Thus information on tumour DNA ploidy and serial CEA measurements might facilitate early detection of recurrence (Rognum, 1986).

Clinicopathological staging remains the most significant variable in the assessment of prognosis in patients with large bowel carcinoma. Flow cytometric determination of tumour DNA ploidy might, however, add valuable clinical information in Dukes' stage C patients.

Supported by The Norwegian Cancer Society. We are indebted to Mrs Liv Lie of The Norwegian Cancer Registry for conscientious assistance.

### References

ABE, S., MAKIMURA, S., ITABASHI, K., NAGAI, T., TSUNETA, Y. & KAWAKAMI, Y. (1985). Prognostic significance of nuclear DNA content in small cell carcinoma of the lung. *Cancer*, **56**, 2025.

ARMITAGE, N.C., ROBINS, R.A., EVANS, D.F., TURNER, D.R., BALDWIN, R.W. & HARDCASTLE, J.D. (1985). The influence of tumour cell DNA abnormalities on survival in colorectal cancer. *Br. J. Surg.*, **72**, 828.

ASHLEY, J.B. (1978). *Evans' histological appearance of tumors*. 3rd. ed. Livingstone: Edinburgh.

ATKIN, N.B. (1976). Prognostic significance of ploidy level in human tumours. I. Carcinoma of the uterus. *J. Natl Cancer Inst.*, **56**, 909.

ATKIN, N.B. & KAY, R. (1979). Prognostic significance of modal DNA value and other factors in malignant tumours, based on 1,465 cases. *Br. J. Cancer*, **40**, 210.

FINAN, P.J., QUIRKE, P., DIXON, M.F., DYSON, G.R., GILES, C.R. & BIRD, C.C. (1986). Is DNA aneuploidy a good prognostic indicator in patients with advanced colorectal cancer? *Br. J. Cancer*, **54**, 327.

FRANKFURT, O.S., SLOCUM, H.K., RUSTUM, Y.M. & 6 others (1984). Flow cytometric analysis of DNA aneuploidy in primary and metastatic human solid tumours. *Cytometry*, **5**, 71.

FRIEDLANDER, M.L., HEDLEY, D.W. & TAYLOR, I.W. (1984). Clinical and biological significance of aneuploidy in human tumours. *Br. J. Cancer*, **37**, 961.

GÖHDE, W. & DITTRICH, W. (1971). Impulsfluorometrie, ein neuartiges Durchflussverfahren zur ultraschnellen Mengenbestimmung von Zellinhaltstoffen. *Acta Histochem.*, **10** (Suppl.), 429.

KOKAL, W., SHEIBANI, K., TERZ, J. & HARADA, R. (1986). Tumor DNA content in the prognosis of colorectal carcinoma. *J. Am. Med. Assoc.*, **255**, 3123.

MANTEL, N. (1966). Evaluation of survival data and two new rank order statistics arising in its consideration. *Cancer Chemoter. Rep.*, **50**, 163.

LAERUM, O.D. & FARSUND, T. (1981). Clinical application of flow cytometry. A review. *Cytometry*, **2**, 1.

MELAMED, M.R., ENKER, W.E., BANNER, P., JANOV, A.J., KESSLER, G. & DARZYNKIEWICZ, Z. (1986). Flow cytometry of colorectal carcinoma with three-year follow up. *Dis. Colon. Rectum.*, **29**, 184.

PEREZ, D.J., TAYLOR, I.W., MILTHORPE, B.K., McGOVERN, V.J. & TATTERSAL, M.H.N. (1981). Identification and quantitation of tumour cells in cell suspension: A comparison of cytology and flow cytometry. *Br. J. Cancer*, **43**, 526.

PETERSEN, S.E., LORENTSEN, M. & BICHEL, P. (1981). A mosaic subpopulation structure of human colorectal carcinomas demonstrated by flow cytometry. *Acta Pathol. Scand. (A)*, **274** (Suppl.), 412.

QUIRKE, P., DYSON, J.E.D., DIXON, M.F., BIRD, C.C. & JOSLIN, C.A.F. (1985). Heterogeneity of colorectal adenocarcinomas evaluated by flow cytometry and histopathology. *Br. J. Cancer*, **51**, 99.

QUIRKE, P., DIXON, F., CLAYDEN, A.D., DYSON, J.E.D., WILLIAMS, N.S. & BIRD, C.C. (1987). Prognostic significance of DNA aneuploidy and cell proliferation in rectal adenocarcinomas. *J. Pathol.*, **151**, 285.

ROGNUM, T.O., THORUD, E., ELGJO, K. & 4 others (1981). DNA flow cytometry (FCM) in carcinomas of the large bowel compared with the two functional cell markers secretory component (SC) and carcinoembryonic antigen (CEA), the histological tumour grade and the clinical stage. *Acta. Pathol. Scand. (A)*, **274** (Suppl.), 417.

ROGNUM, T.O., THORUD, E., ELGJO, K., BRANDTZAEG, P., ØRJASAETER, H. & NYGAARD, K. (1982). Large-bowel carcinomas with different ploidy, related to secretory component, IgA, and CEA in epithelium and plasma. *Br. J. Cancer*, **45**, 921.

ROGNUM, T.O., BRANDTZAEG, P. & THORUD, E. (1983). Is heterogeneous expression of HLA-DR antigens and CEA along with DNA-profile variations evidence of phenotypic instability and clonal proliferation in human large bowel carcinomas? *Br. J. Cancer*, **48**, 543.

ROGNUM, T.O. (1986). A new approach in carcinoembryonic antigen-guided follow-up of large-bowel carcinoma patients. *Scand. J. Gastroenterol.*, **21**, 641.

ROGNUM, T.O., HEIER, H.E., ØRJASAETER, H., THORUD, E. & BRANDTZAEG, P. (1986). Comparison of two CEA assays in primary and recurrent large bowel carcinoma with different DNA ploidy pattern. *J. Cancer Clin. Oncol.*, **22**, 1165.

ROGNUM, T.O., THORUD, E., BRANDTZAEG, P. & 4 others (1987). Plasma CEA in large bowel carcinoma: Which patients should be followed by regular postoperative measurements? Preliminary follow-up results in 100 patients with different tumour DNA-ploidy patterns. *Cancer Detect. Prevent.*, **10**, 347.

STEEL, G.G. (1977). *Growth kinetics of tumours*. Clarendon Press: Oxford, p. 202.

THORUD, E., FOSSÅ, S.D., VAAGE, S. & 4 others (1986). Primary breast cancer. Flow cytometry DNA pattern in relation to clinical and histopathological characteristics. *Cancer*, **57**, 808.

TRIBUKAIT, B., HAMMARBERG, C. & RUBIO, C. (1983). Ploidy and proliferation patterns in colorectal carcinomas related to Dukes' classification and to histological differentiation. *Acta Pathol. Scand., (A)*, **91**, 89.

TURNBULL, R.B., KYLE, K., WATSON, F.R. & SPRATT, J. (1967). Cancer of the colon: The influence of the no-touch isolation technic on survival rates. *Ann. Surg.*, **166**, 420.

VALET, G., RUSSMANN, L. & WIRSCHING, R. (1984). Automated flow-cytometric identification of colo-rectal tumour cells by simultaneous DNA, CEA-antibody and cell volume measurements. *J. Clin. Chem. Clin. Biochem.*, **22**, 935.

VOLM, M., DRINGS, P., MATTERN, J., SONKA, J. VOGT-MOYKOPF, I. & WAYSS, K. (1985*a*). Prognostic significance of DNA patterns and resistance-predictive tests in non-small cell lung carcinoma. *Cancer*, **56**, 1396.

VOLM, M., BRÜGGEMAN, A., GÜNTHER, M., KLEINE, W., PFLEIDERER, A. & VOGT-SCHADEN, M. (1985*b*). Prognostic relevance of ploidy, proliferation, and resistance-predictive tests in ovarian carcinoma. *Cancer Res.*, **45**, 5180.

WOLLEY, R.C., SCHREIBER, K., KOSS, L.G., KARAS, M. & SHERMAN, A. (1982). DNA distribution in human colon carcinomas and its relationship to clinical behaviour. *J. Natl Cancer Inst.*, **69**, 15.